# Acute Severe Lower Gastrointestinal Bleeding in Low- and Medium-Income Countries: An Approach to Management of Two Cases and the Need for Local Guidelines

**DOI:** 10.7759/cureus.26169

**Published:** 2022-06-21

**Authors:** Adedire T Adenuga

**Affiliations:** 1 Surgery, Cedarcrest Hospitals, Abuja, NGA

**Keywords:** acute severe gi bleeding in lmic, colonic bleeding in lmic, management of lower gi bleeding, management of colonic bleeding, diverticular bleeding, colonic bleeding

## Abstract

Acute severe lower gastrointestinal bleeding (LGIB) refers to continued significant bleeding that occurs within the first 24 hours of admission and may be associated with hemodynamic instability. Patients at risk of severe LGIB include elderly patients often with comorbidities and on antiplatelets/anticoagulants.

The accepted guidelines and recommendations used in the management of patients with acute severe LGIB are mainly based on research and evidence from high-income countries which may not be practical in low- and middle-income countries (LMICs). The management of these patients in LMICs is often based on more pressing concerns such as availability of relevant equipment, affordability of care, and accessible technical expertise. In LMICs, surgery plays a major role in patients with severe bleeding and hemodynamic instability refractory to resuscitation and blood transfusion. Here, we discuss the management of two patients who presented with acute severe LGIB and the applicability of the current guidelines in the management of LMIC patients.

## Introduction

Lower gastrointestinal bleeding (LGIB) has been recently defined as colonic and rectal bleeding below the ileocecal valve [1,2]. Patients with overt symptoms present with bright or dark red blood per rectum, clots, or blood mixed in the stool. Acute overt LGIB accounts for 20% of all gastrointestinal bleeding frequently leading to hospital admission, invasive diagnostic evaluation, and treatment [3]. Approximately 85% of these bleeds resolve spontaneously without the need for hospitalization [3]. Acute severe LGIB is defined as continued bleeding within the first 24 hours of hospitalization with a drop in hemoglobin of at least 2 g/dL and/or transfusion requirement of at least two units of packed red blood cells [4].

Predictors of severe colonic bleeding include aspirin use, at least two comorbid illnesses, a pulse of >100 beats/minute, systolic blood pressure of <115 mmHg, transfusion, re-bleeding, 20% reduction in hematocrit, age of >75 years, creatinine of >150 µmol/L, and albumin of <30 g/L [3,5,6].

The specific problems of low- and middle-income countries (LMICs) in the management of patients with acute severe LGIB include absent documented management guidelines and gastrointestinal bleed pathways, lack of advanced imaging, endoscopy, round-the-clock interventional radiology coverage, and inadequate blood transfusion services. Most of the guidelines for the management of gastrointestinal bleeding originate from research drawn from patients in high-income countries (HICs) and may not apply to LMIC patients. Furthermore, LMIC patients present with unusual characteristics compared to patients from HICs because our patients typically present late with worse physiological status and often after the failure of local remedies.

This paper presents two cases of acute severe LGIB and their management and discusses current guidelines and their applicability in the care of LMIC patients.

## Case presentation

Case one

A 72-year-old gentleman presented to our service with dizziness following four episodes of passing bright red blood per rectum which started about three hours prior to admission. He had no abdominal pain, no previous similar episodes, and no bleeding from other body parts. He was hypertensive on amlodipine 10 mg and aspirin 75 mg.

On examination, he was pale, pulse rate was 70 beats/minute, blood pressure was 108/67 mmHg, and respiratory rate was 24 breaths/minute. He had an obese abdomen with no palpable masses or demonstrable ascites. Digital rectal examination revealed no mass, with the examining finger stained with bloody stool. Admitting laboratory values are shown in Table 1. The calculated Oakland score was 26 (20-28% prediction of safe discharge).

**Table 1:** Blood investigation findings at the time of admission.

Test	Patient value	Reference range
Hemoglobin level (g/dL)	8.7	12.5–17.5
White cell count (/µL)	8.2 × 10^3^	3–10 × 10^3^
Platelet count (/L)	112 × 10^9^	150–350 × 10^9^
International normalized ratio	1.2	0.9–1.3

He was immediately resuscitated with fluids and transfused with two pints of blood. A urethral catheter was passed to monitor urine output. A nasogastric tube was inserted and returned clear effluent. Shortly after the second pint of blood was transfused, he had an episode of bleeding per rectum which was estimated at about 1 L. He immediately became unresponsive and hypotensive. Subsequently, three more pints of blood were transfused but his hypotension persisted. He was immediately scheduled for laparotomy, and, intraoperatively, he had a grossly dilated large bowel filled with blood and numerous diverticula extending from the cecum to the proximal sigmoid colon. He underwent a subtotal colectomy with primary anastomosis (ileosigmoid). A total of 13 pints of whole blood and six pints of fresh frozen plasma were transfused perioperatively. The postoperative period was turbulent as he developed respiratory distress on the third postoperative day consistent with adult respiratory distress syndrome requiring re-intubation and mechanical ventilation. He also developed cardiac arrhythmias requiring electrical and medical cardioversion. He suffered a cardiac arrest on postoperative day 10. He was resuscitated and had return of spontaneous cardiac activity after cardiopulmonary resuscitation but suffered a hypoxic brain injury. He was discharged to a neurorehabilitation center after three weeks of surgery.

Case two

A 69-year-old man, known hypertensive, presented at the hospital with a history of passing six episodes of bloody stool. The stool was initially dark in color but later became frank blood with the passage of clots. He gave an estimate of about 1.5 L loss. He had a history of similar episodes of overt bleeding per rectum which were managed with blood transfusions. These episodes had occurred three times over the past five years. He had no change in his bowel habits, no abdominal mass, fever, jaundice, or bleeding from any other body parts. He underwent an emergency percutaneous cardiac intervention for acute chest pain six weeks prior to presentation and was placed on dual antiplatelets (aspirin 75 mg and ticagrelor 90 mg).

On admission, he was mildly dehydrated, with a pulse rate of 104 beats/minute and a regular rhythm. His blood pressure was 118/78 mmHg and hemoglobin was 11.4 g/dL which dropped to 8.5 g/dL after initial resuscitation. His Oakland score was 17 (67-72% prediction of safe discharge).

He was admitted and placed on intravenous fluids and blood transfusion, and a urethral catheter was passed. A nasogastric tube was passed which returned bilous effluent. He was on the second pint of blood transfusion when he developed hypotension and had a cardiac arrest. Immediate cardiopulmonary resuscitation was commenced, and after 15 minutes, he had return of spontaneous cardiac activity. Blood transfusion was continued, and he was moved to the operating theater for laparotomy. Intraoperatively, he had diverticulosis extending from the cecum to the descending colon. He had adhesions of the entire left colon to the small bowel and omentum which may be evidence of previous resolved diverticulitis. Due to his dual antiplatelets, there was copious bleeding from all raw surfaces, including the midline wound and the visceral surface after mobilization of the right and left colon. A subtotal colectomy was performed along with primary ileosigmoid anastomosis. A total of 15 pints of whole blood were transfused and six pints of fresh frozen plasma were administered during the perioperative period. He recommenced his dual antiplatelets on postoperative day two and was discharged home on postoperative day eight.

## Discussion

Although most acute overt LGIB stop bleeding spontaneously and have good outcomes, there is a higher risk of morbidity and mortality in elderly patients with comorbidities and on anticoagulants [7]. In HICs, diverticulosis is regarded as the most common cause of LGIB while bleeding from hemorrhoids predominates in LMICs [7-10]. Severe bleeding from hemorrhoids is generally uncommon, and brisk LGIB should not be attributed to hemorrhoids until other causes have been ruled out [7]. At presentation, resuscitation and initial evaluation of patients should occur concurrently. A proper history to determine the extent of bleeding, comorbidity, and drug history is crucial. In patients with hematochezia and hemodynamic instability, an upper gastrointestinal (UGI) source should always be considered. An esophagogastroduodenoscopy is recommended and where this is not readily available, due to logistic problems or lack of expertise, a nasogastric tube can be passed. The presence of bile in the aspirate suggests a low likelihood of UGIB [7]. Both of our patients had nasogastric tubes passed without aspiration of blood which reduced the suspicion of the UGI being the source of the bleeding.

In LMICs, preoperative localization of bleeding is difficult due to the paucity of sophisticated imaging, advanced endoscopy services, and interventional radiology. Colonoscopy may be impractical in a hemodynamically unstable patient, and visualization may be poor in an unprepared bowel [2]. Most LMIC hospitals do not run a 24-hour gastrointestinal endoscopy service, and patients presenting out of routine work hours may not have access to endoscopy. There is also a general lack of expertise and consumables needed for therapeutic intervention. Both patients in this series did not have gastrointestinal endoscopy as they presented with severe hemodynamic instability.

Regarding surgical options, segmental colonic resection following precise localization of bleeding is the ideal procedure while subtotal colectomy is reserved for patients who are actively bleeding from an unknown source. Blind segmental resection is contraindicated as it is associated with a high chance of rebleeding (up to 75%) with a high rate of morbidity (83%) and mortality (60%) [11,12]. If localization is not possible, subtotal colectomy is performed with a rebleeding rate of about 4% and mortality rate of 7% [12].

During surgery, the surgeon often encounters bleeding, which may be profuse, from the main wound and raw areas following mobilization of the colon. Significant intraoperative blood loss may occur as these patients have coagulopathy which may be associated with hypothermia and thrombocytopenia from massive blood transfusion coupled with the active effect of anticoagulants/antiplatelets used for their comorbid conditions. In patients on vitamin k antagonists, prothrombin complex concentrate is the recommended blood product to be used [13]. Blood products are scarce in LMICs and fresh whole blood may be the only available option.

Many popular guidelines in the management of acute severe LGIB are impracticable in LMICs. The Oakland score predicts safe discharge in patients with LGIB using age, sex, previous LGIB admission, digital rectal findings, heart rate, systolic blood pressure, and hemoglobin level. Patients with a score of 8 or lower are low-risk patients for rebleeding and have a 95% chance of safe discharge [13]. Low hemoglobin levels are associated with higher Oakland scores. Typically, patients in LMICs have lower hemoglobin levels than their HIC counterparts (with normal hemoglobin values as low as 6 g/dL in some African countries) [14,15]. Although both patients had scores >8 on admission which precluded safe discharge, LMIC patients are likely to get higher scores de novo even without severe bleeding, and this scoring would require local validation for its use.

The European Society for Gastrointestinal Endoscopy (ESGE), British Society of Gastroenterology (BSG), and American College of Gastroenterology (ACG) recommend colonoscopy (and therapeutic intervention) in hemodynamically stable patients and the use of angiography and transcatheter arterial embolization in hemodynamically unstable patients [7,13,16]. The ESGE recommends the use of computed tomography angiography for patients with hemodynamic instability to localize bleeding and the use of four-factor prothrombin complex concentrate (PCC) [13]. These recommendations are not practical in LMICs as most hospitals do not run a 24-hour colonoscopy service, and where this service is available, there is often a lack of consumables and skilled endoscopists required to perform hemostatic procedures endoscopically. Sophisticated imaging is also lacking as well as a general lack of trained interventional radiologists. Blood transfusion services are still rudimentary and massive blood transfusion is often with whole blood as blood products such as platelet concentrate, PCC, fresh frozen plasma, and packed red blood cells may not be readily available. Lack of specialists and defective healthcare systems also imply that there is no gastrointestinal bleeding lead and no documented definite pathways of management of LGIB.

In patients with acute severe LGIB with hemodynamic instability despite blood transfusion, these guidelines downplay the role of surgery and emphasize treatment with interventional radiology services and endoscopic intervention which are not readily available in LMICs. The various guidelines have been designed in HICs and have not been validated locally. Guidelines native to LMICs need to be developed according to the level of accessible technical expertise, the available imaging present, and the level of blood transfusion support.

A consensus meeting of gastrointestinal experts practicing in LMICs is required to draft proper management protocol and guidelines for our patients based on local data and evidence. Some suggestions would include recommendation of initial admission of patients who report significant bleeding per rectum and stratification of risk using a simple tool such as the shock index. Patients should be stratified as being unstable (shock index >1) or stable (shock index <1) [7]. As much as possible, especially in patients requiring massive blood transfusion, fresh whole blood should be given in the absence of blood products. Although old blood may be used in the initial phases, efforts should be made to obtain fresh whole blood. In patients with acute severe LGIB, a UGI source of bleeding should be ruled out. In areas without a readily available endoscopy service, a nasogastric tube can be passed, and aspiration of bile would reduce the suspicion of an UGI source of bleeding [7]. Furthermore, in the absence of an endoscopist with relevant expertise skilled at hemostatic colonoscopic techniques, preoperative colonoscopy in acute severe bleeding should not be encouraged. In the setting of continued bleeding with associated persistent hemodynamic instability despite fluid and hemostatic resuscitation, these patients should be advised for surgical intervention. The option of surgical intervention, as most patients would not have preoperative localization due to earlier stated limitations, should be a subtotal colectomy with either a primary anastomosis or formation of an ileostomy. Blind segmental resection should be strongly discouraged as it is associated with an unacceptably high rate of rebleeding and mortality [11,12].

More local data are needed on patients with acute severe LGIB along with practical guidelines in the management of these patients suiting the peculiarities of our patients and the current healthcare system.

## Conclusions

Acute severe LGIB with hemodynamic instability poses a challenge in the management in LMICs. The current guidelines in the management of acute severe LGIBs may not be entirely applicable in our setting due to the lack of sophisticated equipment, manpower, and established health systems, and surgery plays a big role in the management of these patients. Evidence-based local guidelines based on availability and affordability should be generated and treatment pathways outlined.
